# The prognostic value of expression of HIF1α, EGFR and VEGF-A, in localized prostate cancer for intermediate- and high-risk patients treated with radiation therapy with or without androgen deprivation therapy

**DOI:** 10.1186/1748-717X-7-66

**Published:** 2012-04-30

**Authors:** Damien C Weber, Jean-Christophe Tille, Christophe Combescure, Jean-François Egger, Mahomet Laouiti, Karim Hammad, Perrine Granger, Laura Rubbia-Brandt, Raymond Miralbell

**Affiliations:** 1 Department of Radiation Oncology, Geneva University Hospital, Geneva, Switzerland; 2 Division of Clinical Pathology, Geneva University Hospital, Geneva, Switzerland; 3 Department of Biostatistics and Clinical Epidemiology, Geneva University Hospital, Geneva, Switzerland; 4 Unilabs Cytopath, CH-1227, Carouge, Switzerland; 5 Radiation Oncology Department, Geneva University Hospital, CH-1211, Geneva 14, Switzerland

## Abstract

**Purpose:**

Androgens stimulate the production of hypoxia-inducible factor (HIF1α) and ultimately vascular endothelial growth factor (VEGF-A). Additionally, epithelial growth factor (EGF) mediates HIF1α production. Carbonic anhydrase IX (CAIX) expression is associated with tumor cell hypoxia in a variety of malignancies. This study assesses the prognostic relation between HIF1α, VEGF-A, EGF Receptor and CAIX expression by immunochemistry in diagnostic samples of patients with intermediate- and high-risk localized prostate cancer treated with radiation therapy, with or without androgen deprivation therapy (ADT).

**Materials and methods:**

Between 1994 and 2004, 103 prostate cancer patients (mean age, 68.7 ± 6.2), with prostate cancer (mean PSA, 13.3 ± 3.7), were treated with radiation therapy (RT, median dose, 74 Gy). Fifty seven (55.3%) patients received ADT (median duration, 6 months; range, 0 – 24). Median follow-up was 97.6 months (range, 5.9 – 206.8).

**Results:**

Higher EGFR expression was significantly (*p* = 0.04) correlated with higher Gleason scores. On univariate analysis, HIF1α nuclear expression was a significant (*p* = 0.02) prognostic factor for biological progression-free survival (bPFS). A trend towards significance (*p* = 0.05) was observed with EGFR expression and bPFS. On multivariate analysis, low HIF1α nuclear (*p* = 0.01) and high EGFR (*p* = 0.04) expression remained significant adverse prognostic factors.

**Conclusions:**

Our study suggests that high nuclear expression of HIF1α and low EGFR expression in diagnostic biopsies of prostate cancer patients treated with RT ± ADT is associated with a good prognosis.

## Background

It has been recognized that cancer-stromal cell interactions is a major player of malignant behavior in cancer. More specifically, hypoxia may trigger vascular endothelial growth factor (VEGF) expression via the transcription complex of hypoxia-inducible factor HIF1α. Hypoxia and the consequential angiogenesis may play a major role in prostate cancer progression, as VEGF and HIF1α is increased in prostate cancer, when compared to benign prostatic hypertrophy [1,2]. Additionally, a direct link between androgen receptors and pro-angiogenic factors may exist, as HIF1α, via epithelial growth factor (EGF), expression is increased with androgens [3] and decreased in prostatectomy specimen treated with pre-operative androgen deprivation therapy (ADT) [4]. The carbonic anhydrase IX (CAIX) gene is a target of HIF1α and is up-regulated in hypoxia [5]. Likewise, it has been shown that androgen deprivation in cell culture decreases VEGF mRNA expression [6] and castration in rodents’ decreases VEGF expression in androgen sensitive xenografts [7]. The prognostic significance of EGF-receptor (EGFR), HIF1α and VEGF-A in prostate cancer is somewhat disputed. Some series have shown a negative outcome in patients with pre-treatment VEGF expression [8], whilst other studies have suggested an improved outcome in patients undergoing radical prostatectomy [4].

Given the lack of strong prognostic evidence regarding these markers in patients who may benefit from dose escalation RT, we analyzed tumor expression of EGFR, HIF 1α, VEGF-A and CAIX with respect to freedom from biochemical progression in men with intermediate- and high-risk prostate cancer treated with RT, with or without ADT.

## Methods

Between 1994 and 2004, 103 patients with clinically localized (according to the 2002 TNM American Joint Committee on Cancer staging system: cT1c – cT2b) or locally advanced (cT3a – cT4) non-metastatic prostate cancer with diagnostic samples with histological proven adenocarcinoma were indentified in our institutional database. All patients underwent bone scintigraphy and endo-rectal MRI staging prior to radiation therapy (RT). All patients were classified as having intermediate-risk disease (*n* = 53; 51.5%) or high risk (*n* = 50; 48.5%), as defined by d’Amico *et al.* criteria (Table 1). Six months ADT was administered to 57 (55.3%) patients (Table 1). RT (mean dose, 75.1 ± 2.8 Gy; Table 1) was delivered concomitantly after 3 months of ADT for those receiving ADT or immediately for those not receiving ADT. Patients were followed with 6-monthly PSA tests. This study was approved by the institutional ethic committee (NAC 08-076R) and complied to the Helsinki declaration. Prior to study initiation, written, informed consent to perform this analysis was obtained from all patients. The mean duration of the follow up time was 96.4 ± 33.7 months. No patients were lost to follow-up.

**Table 1:** Intermediate- and high-risk prostate cancer patient’s and treatment characteristics

Number of patients	103
Age (years)	
Median	69.1
Range	56.0 – 81.0
T stage	
T1	27 (26.2%)
T2	24 (23.3%)
T3	51 (49.5%)
T4	1 (1.0%)
Gleason score	
<7	60 (58.2%)
7	40 (38.8%)
>7	2 (2.0%)
Unknown	1 (1.0%)
PSA (ng/mL)	
Median	13.1
Range	3.1 – 20.0
Risk category	
Intermediate-risk	53 (51.5%)
High-risk	50 (48.5%)
Dose (Gy)	
Median	74.0
Range	64.4 – 78.4
ADT	
Yes	57 (55.3%)
No	46 (44.7%)

*PSA* prostatic-specific antigen ; *ADT* anti-androgen deprivation therapy.

### Immunochemistry

All tissues obtained by prostate biopsy or transurethral resection of the prostate were formalin-fixed or Duboscq-Brazil-fixed and paraffin embedded. The hematoxylin-eosin stained sections were reviewed to confirm the diagnosis and only sections showing typical Gleason score were selected. For immunohistochemistry (IHC), section 4 μm from one representative block of each patient were deparaffinized, rehydrated, and then submitted to IHC analysis as follow.

#### HIF1α

After boiling with a pressure cooker in Tris-EDTA pH:9.0 buffer for 3 min, sections were incubated in DAKO autostainer with the monoclonal mouse HIF1α antibody clone H1alpha67 (NB100-123, NOVUS Biologicals) diluted 1/1000 and stained with CSA-II-Biotin-free Tyramide Signal Amplification System (K1497, DAKO). Renal clear cell carcinoma served as a positive control. Primary antibody was substituted with mouse IgG2b for negative control.

#### VEGF-A

After boiling with a pressure cooker in citrate pH:6.0 buffer for 3 min, sections were incubated in DAKO autostainer with the monoclonal mouse VEGF-A antibody clone VG1 (18–7328, ZYMED Laboratories) diluted 1/50 and stained with stained with EnVision anti mouse/rabbit (K5007, DAKO). Renal clear cell carcinoma served as a positive control. Primary antibody was substituted with mouse IgG1 for negative control.

#### EGFR

After proteinase K (S3020, DAKO) digestion for 30 min, only for formalin-fixed tissu, sections were incubated with the monoclonal mouse EGFR antibody clone 31 G7 (28–0005, ZYMED Laboratories) diluted 1/20 and stained with EnVision anti mouse/rabbit (K5007, DAKO). Lung adenocarcinoma served as a positive control. Primary antibody was substituted with mouse IgG1 for negative control.

#### CAIX

After boiling with a pressure cooker in citrate pH:6.0 buffer for 3 min, sections were incubated with the polyclonal rabbit CA-IX antibody (NB100-417, NOVUS Biologicals) diluted 1/1500 and stained with EnVision anti mouse/rabbit (K5007, DAKO). Renal clear cell carcinoma served as a positive control. Primary antibody was substituted with nonimmune rabbit immunoglobulin (DAKO) for negative control. Visualization of the primary antibody was achieved using diaminodenzine as chromogen and section were lightly counterstained with hematoxylin.

### Quantification

The percentage and intensity of positively nuclear and intensity of cytoplasmic staining in tumor cells were evaluated. HIF1α expression was assessed in tumor cells using a modified previously published semiquantitative scoring [9]. The immunohistochemical results for HIF1α were classified as follow for nuclear and cytoplasmic percentage staining: 0, no staining; 1, less than 1% of cells; 2, 1–10%; 3, 10–50%; 4, more than 50%; for nuclear and cytoplasmic intensity staining [8]: 0, no staining; 1, weak staining; 2, moderate staining; 3, strong staining. The percentage and intensity nuclear and cytoplasmic intensity scores were added together to give a final immunoreactive score (IRS) of 0 to 10. HIF1α was categorized as low HIF1α = ≤ 50% cells staining and high HIF1α = > 50% cells staining.

VEGF-A expression was assessed in tumor cells using a previously published semiquantitative scoring in prostate tissue [10]. The percentage of positively tumor cells was evaluated and the VEGF-A staining intensity was assessed. The percentage and intensity scores were added together to give a final immunoreactive score (IRS) of 0 to 8. VEGF-A IRS scores were categorized as low VEGF-A = IRS score < 5, high VEGF-A = IRS score >5.

EGFR expression was assessed in tumor cells using a previously published semiquantitative scoring in prostate tissue [11]. EGFR expression was assessed in tumor cells and only membranous EGFR staining was considered. The percentage of positive tumor cells was estimated as follow: 0, no membranous staining, 1, <30% of cells; 2, 30–50% of cells; 3, >50% of cells. The staining intensity was scored as follow: 0, no staining; 1, weak staining; 2, moderate staining; 3, strong staining. Tumor were subsequently categorized as negative (no membranous staining), strongly positive (>50% with moderate intensity or >30% of cells with strong intensity) or weakly positive if not reaching the criteria selected above.

CA-IX expression was assessed in tumor cells using a previously published score [12]. Only membranous CA-IX staining was considered. Immunostaining of >10% of tumor cells was necessary to be positive.

Diagnostic biopsies were assessed by a two prostate-cancer histopathologist blinded to patient outcome.

### Statistical considerations

Biochemical progression-free survival (bPFS), cancer-specific (CSS) and overall survival (OS) were calculated from the date of RT using Kaplan-Meier estimates. The events were death (all causes of death included) for OS, death from prostate cancer for CSS and biochemical PSA failure or death for bPFS. Biochemical failure was defined by use of the Houston criteria. Patients free from biochemical failure were censored on the date of their last PSA test. Proportions were compared using the Chi-square test for values > 5 and Fisher’s exact test for values ≤ 5. Differences between groups were assessed using the log-rank test. The log-rank test was used to compare different survival functions according to the HIF1α, EGFR and VEGF-A expression. Multivariate cause-specific Cox models that accounted for competing risks were fit separately for prostate cancer patients. The proportional hazards assumption was tested using scaled Schoenfeld residuals, with visual inspection of the log minus log plots. Predictors included PSA, Gleason, age, HIF1α, EGFR and VEGF-A expression. All statistical tests were two sided, with alpha levels lower than .05 considered statistically significant.

## Results

Median follow-up was 8.1 years and 27 (26.2%) patients died, 8 of prostate cancer. The estimated 8-year CSS and OS was 70.4% (95%CI: 47.3 – 93.5) and 81.4 (95%CI: 73.2 –89.4), respectively. Twenty-nine (28.2%) patients developed biochemical failure. The estimated 8-year bPFS was 73.4% (95%CI: 64.0 – 82.8). No CAIX expression was observed in this series, whereas the majority of tumors had a strong HIF1α and VEGF-A expression (Table 2).

**Table 2:** **Distribution of EGFR, HIF1α,VEGF-A and CAIX for prostate cancer patients ( *n* ** **= 103) treated with RT, with or without ADT**

	*n* (%)
EGFR	
negative	42(40.8)
weak	35 (34.0)
strong	26 (25.2)
HIF1α (nuclear staining)	
No staining	9 (8.7)
< 10%	3 (2.9)
10 – 50%	9 (8.7)
> 50	81 (78.6)
NA	1 (1)
VEGF-A	
low	27 (26.2)
high	76 (73.8)
CA-IX	
negative	103 (100.0)
positive	0 (0)

*RT* radiotherapy; *ADT* anti-androgen deprivation therapy; *NA* not assessable.

A significant correlation was noted between higher EGFR expression and higher Gleason score (*p* = 0.04). No, weak and strong EGFR expression was observed in 30 (50.0%), 16 (26.7) and 14 (23.3%) in tumor with Gleason scores < 7, respectively. The corresponding values were 11 (26.2%), 19 (45.2%) and 12 (28.6%) in tumor with Gleason scores ≥ 7, respectively. A trend toward significance was observed between higher EGFR expression and risk categories (*p* = 0.07). No, weak and strong EGFR expression was observed in 24 (50.0%), 11 (22.9%) and 13 (27.1%) in intermediate-risk tumors. The corresponding values were 18 (32.7%), 24 (43.6%) and 13 (23.6%) in high-risk tumors. Conversely, no significant association was observed with EGFR expression and PSA (*p* = 0.27). No significant correlation was also observed between HIF1α and VEGF-A expression and any of the baseline clinical characteristics (Gleason, PSA and risk category).

**Figure 1:**
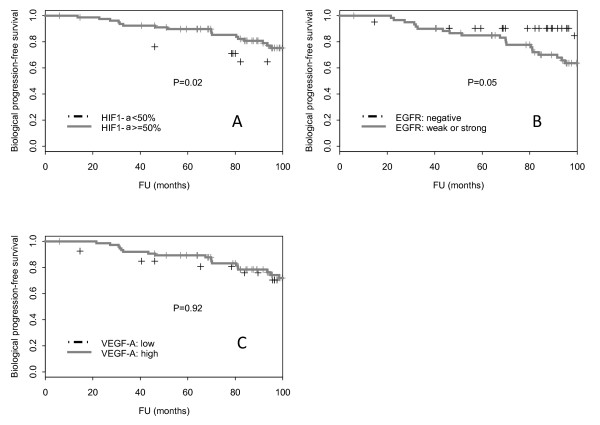
Biological progression-free survival as a function of HIF1α (A), EGFR (B) and VEGF-A (C) expression in 103 intermediate- and high-risk prostate cancer.

Univariate analysis of the parameters in relation to biochemical control is detailed in Table 3. High expression of HIF1α was associated with a significant increase in bPFS (*p* = 0.019; Table 3). The 8-year bPFS was 75.5% [95%CI: 65.1 – 85.9] and 64.6% [95%CI: 43.0 – 86.2] for patients with > 50% and ≤ 50% nuclear expression, respectively (Figure 1). A statistical trend was observed with expression of EGFR: strong IHC expression was a predictor of a shorter time to biological failure (*p* = 0.05, Table 3; Figure 1). The 8-year bPFS was 63.7% [95%CI: 50.8 – 76.6], and 90.4% [95%CI: 81.4 – 99.4] for patients with strong/weak and no EGFR staining, respectively (Figure 1). VEGF-A was however not correlated with biological outcome (*p* = 0.92; Table 3). The 8-year bPFS was 70.5% [95%CI: 51.7 – 89.3] and 74.4% [95%CI: 63.6 – 85.2] for patients with low and high VEGF-A staining, respectively. PSA (*p* = 0.41), ADT (*p* = 0.22), Gleason (*p* = 0.50), Risk category (*p* = 0.58), age (*p* = 0.13) and dose (*p* = 0.78) were not significant predictors of bPFS (Table 3). On multivariate analysis, low HIF1α (*p* = 0.01) and high EGFR (*p* = 0.04) expression remained significant adverse prognostic factors (Table 4).

**Table 3:** Univariate analysis of biochemical progression-free survival

**Parameter**	**8-year bPFS [%]** **(**_**95%**_**CI)**	** *p* **
PSA		0.41
≤ 15 ng/ml	66.6 (44.3–88.9)	
>15 ng/ml	75.1 (64.9–85.3)	
ADT		0.22
no		
	66.7 (51.2–82.2)	
yes	78.4	
	(67.0–89.8)	
Dose (prostate)		0.78
≤ 74 Gy	76.7 (64.9–88.5)	
>74 Gy	68.8 (53.5–84.1)	
Gleason		0.50
< 7	75.4 (63.4–75.4)	
≥ 7	72.0 (56.9–87.1)	
Risk category		0.58
Intermediate-risk	76.4 (63.3–89.5)	
High-risk	70.9 (57.8–84.0)	
Age		0.13
≤ 61 years	70.1	
	(59.7–80.5)	
> 61 years	88.9	
	(68.3–100.0)	
HIF1α		0.019
≤ 50%	64.6 (43.0–86.2)	
>50%	75.5 (65.1–85.9)	
EGFR		0.05
strong	61.6 (41.8–81.4)	
weak	65.1 (48.0–82.2)	
negative	85.0 (71.9–98.1)	
VEGF-A		0.92
l ow	70.5 (51.7–89.3)	
high	74.4 (63.6–85.2)	

*PSA* prostatic-specific antigen, *ADT* anti-androgen deprivation therapy.

**Table 4:** Multivariate analysis for biochemical progression-free survival

**Parameter**	**HR** **(95%CI)**	** *p* **	**HR** **(95%CI)**	** *p* **	**HR** **(95%CI)**	** *p* **
PSA		0.19		0.24		0.22
≤ 15 ng/ml	1.0		1.0		1.0	
>15 ng/ml	0.55 (0.23–1.33)		0.59 (0.25–1.42)		0.57 (0.24–1.38)	
Gleason		0.91		0.90		0.74
< 7	1.0		1.0		1.0	
≥ 7	1.04 (0.48–2.27)		0.95 (0.44–2.07)		1.14 (0.53–2.45)	
Age		0.16		0.17		0.16
≤61 years	1.0		1.0		1.0	
>61 years	1.76 (0.80–3.88)		1.73 (0.79–3.79)		1.76 (0.80–3.85)	
HIF1α		0.01				
≤ 50%	1.0					
>50%	0.37 (0.17–0.82					
EGFR				0.04		
negative			1.0			
Weak or strong			(1.03–7.43)			
VEGF-A						0.96
low					1.0	
high					0.97 (0.39–2.42)	

*PSA* prostatic-specific antigen.

ADT administration did not improve the biological outcome of patients with low or high HIF1α expression (Table 5).

**Table 5:** Bio-chemical Progression-free survival in patients treated with exclusive RT and combined RT and ADT as a function of HIF1α, EGFR and VEGF-A expression

**IHC expression**	**No ADT**	**ADT**	** *p* **
	**8-year bPFS (%)[95%CI]**	**8-year bPFS (%)[95%CI]**	
Low-HIF1α †	63.6 [40.7 – 99.5]	70.0 [46.7 – 100.0]	0.46
High-HIF1α ‡	68.0 [52.5 – 88.2]	80.7 [69.4 – 91.9]	0.30
No EGFR	82.2 [67.8 – 99.7]	100.0 [NA]	0.36
Weak EGFR **	37.3 [13.8 – 100.0]	74.1 [58.1 – 94.6]	0.15
Strong EGFR *	62.5 [38.9 – 100.0]	61.5 [40.0 – 94.6]	0.65
low VEGF-A ¶	68.6 [44.5 – 100.0]	74.8 [56.1 – 99.7]	0.33
high VEGF-A ¶	67.8 [45.9 – 84.2]	80.2 [68.0 – 94.7]	0.45

*RT* radiation therapy; *IHC* Immuno-histochemistry expression; *ADT* Androgen deprivation therapy.

† ≤ 50% nuclear.

‡ > 50% nuclear.

* > 50% with moderate intensity or >30% of cells with strong intensity.

**Not reaching the above criteria.

low VEGF-A = IRS score < 5, high VEGF-A = IRS score >5.

## Discussion

In our study, approximately three quarters of all prostate tumors strongly expressed HIF1α, and VEGF-A (Table 2), a number that is similar to the figures reported by others in prostate cancer [8]. Unlike locally-advanced prostate cancer, strong EGFR expression was observed in one quarter of the studied patients, as reported by other investigators in early stage prostate cancer [13].

High expression of HIF1α was unexpectedly associated with an improved biochemical survival (Table 3; Figure 1). These findings contrast with published results suggesting that low expression of this transcription factor is associated with better clonogenic survival in breast cancer cell lines under hypoxia [14] or cervical cancer treated with RT [15], but published reports on these cancers are not unequivocal [16]. In a head and neck (H&N) series, high expression of HIF1α in 79 surgically treated patients with squamous cell carcinoma was significantly associated with improved disease-free and overall survival in multivariate analysis [17]. Likewise, HIF1α expression was assessed in 85 patients with early stage T_1−2_ H&N squamous cell carcinoma treated with surgery alone by IHC on tissue micro arrays [18]. High expression of HIF1α was associated with an improved 5-year disease-free and overall survival in multivariate analysis. Of note, the transcription of the HIF1α-subunit is regulated by two synergistic mechanisms. First, PHD enzymes catalyze the hydroxylation of two prolin residues in the oxygen degradation-dependant domain of this subunit. Consequently, HIF1α will be recognized by von Hippel-Lindau protein that will allow degradation by the proteosome. The second mechanism involves Factor Inhibiting Hypoxia-inducible factor 1 (FIH-1). Under normoxic conditions, FIH-1 hydroxylates an aspirigine residue in the C-terminal portion of the two HIF1α isoforms. This modification prevents the interaction of HIF1α C-terminal domain with the transcriptional co-activator p300, thus decreasing HIF1α transcriptional activity and increasing HIF1α expression. Interestingly, nuclear FIH-1 was associated with a favorable biochemical survival in a recent prostatectomy series [19]. These data suggests that HIF1α may be associated with a better biochemical outcome in prostate cancer patients, although the *r*-value of the FIH-1/HIF1α correlation was not given by the English authors [19]. Noteworthy, HIF1α was not prognostic in prostate cancer patients included in a French dose escalation study [20] and an US prostatectomy series [4] using IHC and gene expression, respectively.

The reasons for these discrepant observations are unclear. Notwithstanding the issue of hypoxia and radio-resistance, the potential phenotypic aggressiveness of HIF1α-negative tumor cells has been documented in series [21]. HIF1α is known to play a role in promoting tumor cell’s apoptosis. In a series of embryonic stem cells model the proliferation HIF1α +/+ knockout cells was reduced or delayed in hypoxic conditions [21]. Conversely, growth of HIF1α −/− knockout embryonic cells was not retarded but was increased, possibly because of decreased hypoxia-induced apoptosis and increased stress-induced proliferation [21]. It may well be that these HIF1α-negative cells may loses their ability to undergo apoptosis, at a distance from blood vessels, reducing thus their critical dependence on vascular supply. Our results contrast radically with those published by the Royal Marsden group (RMH) [8]. In this study, the diagnostic biopsies of 308 localized prostate cancer patients were entered into two sequential dose-escalation trials (64 Gy *vs.* 74 Gy) with ADT. The same biochemical failure definition was used in both studies. Patients in the RMH study has somehow more favorable characteristics when compared to those in the present study (T_2_ 59% *vs.* 23%; Gleason < 7, 74% *vs.* 58%; median PSA, 11.5 *vs.* 13.1 ng/ml). Possible explanation for these contradictory findings may include imbalances between the two cohorts (the biochemical progression rate was 38% *vs.* 28% in the RMH and present series, respectively) or the immunoreactivity assessment methodology. In the RMH study, HIF1α was assessed in terms of cytoplasmic staining. We found HIF1α-nuclear only expression in our study for this nuclear transcription factor (Table 2). Unlike the RMH staining methodology, we used double IHC staining method, as detailed by Vaughan *et al*. [22]. Alternatively, more advanced prostate tumors may express differentially HIF1α. HIF1α mRNA gene expression was significantly unregulated in blood samples of localized prostate cancer patients, when compared to individuals with no malignancies or those with more advanced tumors in a recent prospective study [23].

Strong EGFR expression was associated (*p* = 0.05) with a decrease in bPFS (Table 3; Figure 1). The prognostic relevance of EGFR expression was also observed in a recent Italian series of prostate cancer patients [13]. The observed median time to biochemical failure in this series was 104 and 30 months in EGFR <50% and ≥50% tumors, respectively (HR, 2.5; *p* = 0.02). EGFR expression may have a role in the development of prostate cancer [24]. EGFR is down regulated at the transcriptional level by androgens in normal prostate tissue but up-regulated in prostate malignancy, especially in androgen-independent prostate cancer. Di Lorenzo *et al*., reporting on 76 patients with androgen-dependent and -independent prostate cancer, observed 41%, 76% and 100% EGFR expression in radical prostatectomy, hormone-sensitive and hormone-refractory metastatic patients, respectively [24]. We have observed a significant association between EGFR expression and higher Gleason scores (*p* = 0.04). These results may also be in keeping with other series [24]. It remains to be demonstrated if EGFR therapeutic targeting may optimize patient outcome [25]. EGFR prognostication needs to be more fully assessed in the framework of prospective studies.

The expression of the angiogenic factor VEGF-A, a soluble growth factor acting as a specific endothelial mitogen, and its receptor may be an important factor in the prostate carcinogenesis. In our series, high-VEGF-A expression was usually not associated with biochemical failure (Table 3, Figure 1). The absence of a significant correlation observed in our study may be due to the small sample size, that may have limited the statistical power to detect associations between VEGF-A expression and biochemical outcome, or to the diffuse and multifocal IHC-expression pattern in prostate cancer that may render the quantification of this glycoprotein somewhat difficult [26,27].

CAIX is normally expressed in epithelial cells of the intestines and stomach but may be expressed when tumor cell hypoxia occurs in malignancies. It is expressed in carcinomas derived from cells not expressing this membrane-bound glycoprotein, such as those observed in lung, breast or kidney and may be associated with a negative prognosis in these tumours. Prostate cancer cell line may express CAIX in strong hypoxic conditions. In our series, none of the prostate cancer cells expressed CAIX in diagnostic samples and could thus not be considered strongly hypoxic (Table 2). The NB100-417 antibody, used in this series, has been associated with false positive but not false negative IHC results. As such, prostate cancer cells in core our histological samples were not hypoxic.

We could not demonstrate an impact on biochemical outcome in patients with unfavorable (i.e. tumors low HIF1α and/or high EGFR immunoreactivity) tumors treated with RT ± ADT (Table 5). Androgen deprivation improves tumor oxygenation and may thus increase the efficacy of RT in patients with unfavorable prognosis. Small patient numbers complicate the analysis of these findings. The number of patients in the low-HIF1α group receiving or not receiving ADT was 10 and 11, respectively (data not shown). In our series, a better 8-year bPFS was observed with low HIF1α immunoreactivity treated with ADT when compared to RT alone. Future efforts should be directed toward the understanding of the role of these parameters in selecting treatment for intermediate- and high-risk prostate cancer patients in the frame of prospective studies.

This study has potential limitations inherent in all retrospective analyses, including uncontrolled patients selection into the different treatment groups. Major limitations of this study include but are not limited to the IHC evaluation in a limited sample of diagnostic tissue that may not reflect the intrapatient heterogeneity of tissue marker expression and the limited overall number of patients. To our knowledge, the present report is however the first to report a positive association between biochemical outcome and high-HIF1α immunoreactivity in intermediate- and high-risk prostate cancer patients treated with RT.

## Conclusions

In summary, HIF1α and VEGF-A was frequently expressed in prostate cancer cells. HIF1α possibly non-hypoxia related expression in diagnostic biopsies was associated with an improved biochemical survival. EGFR immunoreactivity was associated with poor outcome.

## Abbreviations

VEGF, Vascular endothelial growth factor; HIF 1α, Hypoxia-inducible factor α; EGF, Epithelial growth factor; ADT, Androgen deprivation therapy; CAIX, Carbonic anhydrase IX; EGFR, EGF-receptor; RT, Radiation therapy; IRS, Immunoreactive score; BPFS, Biochemical progression-free survival; CSS, Cancer-specific survival; OS, Overall survival; RMH, Royal Marsden group.

## Competing interests

The authors declare that they have no competing interests.

## Author’s contribution

DCW and RM were responsible for the primary concept and the design of the study; DCW and JCT, performed the data capture and analysis. DCW drafted the manuscript; DCW and CC performed the statistical analysis; DCW and JCT reviewed patient data; all authors revised the manuscript. All authors have read and approved the final manuscript.

## References

[B1] Li R Younes M Wheeler TM Scardino P Ohori M Frolov A Expression of vascular endothelial growth factor receptor-3 (VEGFR-3) in human prostate Prostate 2004 Feb 1 58 2 193 199 10.1002/pros.10321 14716745

[B2] Zhong H Semenza GL Simons JW De Marzo AM Up-regulation of hypoxia-inducible factor 1alpha is an early event in prostate carcinogenesis Cancer Detect Prev 2004 28 2 88 93 10.1016/j.cdp.2003.12.009 15068831

[B3] Boddy JL Fox SB Han C Campo L Turley H Kanga S The androgen receptor is significantly associated with vascular endothelial growth factor and hypoxia sensing via hypoxia-inducible factors HIF-1a, HIF-2a, and the prolyl hydroxylases in human prostate cancer Clin Cancer Res 2005 Nov 1 11 21 7658 7663 10.1158/1078-0432.CCR-05-0460 16278385

[B4] Mori R Dorff TB Xiong S Tarabolous CJ Ye W Groshen S The relationship between proangiogenic gene expression levels in prostate cancer and their prognostic value for clinical outcomes Prostate 2010 Nov 1 70 15 1692 1700 10.1002/pros.21204 20564320

[B5] Wykoff CC Beasley N Watson PH Campo L Chia SK English R Expression of the hypoxia-inducible and tumor-associated carbonic anhydrases in ductal carcinoma in situ of the breast Am J Pathol 2001 Mar 158 3 1011 1019 10.1016/S0002-9440(10)64048-5 11238049 PMC1850356

[B6] Stewart RJ Panigrahy D Flynn E Folkman J Vascular endothelial growth factor expression and tumor angiogenesis are regulated by androgens in hormone responsive human prostate carcinoma: evidence for androgen dependent destabilization of vascular endothelial growth factor transcripts J Urol 2001 Feb 165 2 688 693 10.1097/00005392-200102000-00095 11176459

[B7] Mukherjee P Sotnikov AV Mangian HJ Zhou JR Visek WJ Clinton SK Energy intake and prostate tumor growth, angiogenesis, and vascular endothelial growth factor expression J Natl Cancer Inst 1999 Mar 17 91 6 512 523 10.1093/jnci/91.6.512 10088621

[B8] Vergis R Corbishley CM Norman AR Bartlett J Jhavar S Borre M Intrinsic markers of tumour hypoxia and angiogenesis in localised prostate cancer and outcome of radical treatment: a retrospective analysis of two randomised radiotherapy trials and one surgical cohort study Lancet Oncol 2008 Apr 9 4 342 351 10.1016/S1470-2045(08)70076-7 18343725

[B9] Zhong H De Marzo AM Laughner E Lim M Hilton DA Zagzag D Overexpression of hypoxia-inducible factor 1alpha in common human cancers and their metastases Cancer Res 1999 Nov 15 59 22 5830 5835 10582706

[B10] Green MM Hiley CT Shanks JH Bottomley IC West CM Cowan RA Expression of vascular endothelial growth factor (VEGF) in locally invasive prostate cancer is prognostic for radiotherapy outcome Int J Radiat Oncol Biol Phys 2007 Jan 1 67 1 84 90 10.1016/j.ijrobp.2006.08.077 17189065

[B11] Schlomm T Kirstein P Iwers L Daniel B Steuber T Walz J Clinical significance of epidermal growth factor receptor protein overexpression and gene copy number gains in prostate cancer Clin Cancer Res 2007 Nov 15 13 22 Pt 1 6579 6584 18006757 10.1158/1078-0432.CCR-07-1257

[B12] Al-Ahmadie HA Alden D Qin LX Olgac S Fine SW Gopalan A Carbonic anhydrase IX expression in clear cell renal cell carcinoma: an immunohistochemical study comparing 2 antibodies Am J Surg Pathol 2008 Mar 32 3 377 382 10.1097/PAS.0b013e3181570343 18300814

[B13] Peraldo-Neia C Migliardi G Mello-Grand M Montemurro F Segir R Pignochino Y Epidermal Growth Factor Receptor (EGFR) mutation analysis, gene expression profiling and EGFR protein expression in primary prostate cancer BMC Cancer 2011 11 31 10.1186/1471-2407-11-31 21266046 PMC3040720

[B14] Blancher C Moore JW Talks KL Houlbrook S Harris AL Relationship of hypoxia-inducible factor (HIF)-1alpha and HIF-2alpha expression to vascular endothelial growth factor induction and hypoxia survival in human breast cancer cell lines Cancer Res 2000 Dec 15 60 24 7106 7113 11156418

[B15] Birner P Schindl M Obermair A Plank C Breitenecker G Oberhuber G Overexpression of hypoxia-inducible factor 1alpha is a marker for an unfavorable prognosis in early-stage invasive cervical cancer Cancer Res 2000 Sep 1 60 17 4693 4696 10987269

[B16] Hutchison GJ Valentine HR Loncaster JA Davidson SE Hunter RD Roberts SA Hypoxia-inducible factor 1alpha expression as an intrinsic marker of hypoxia: correlation with tumor oxygen, pimonidazole measurements, and outcome in locally advanced carcinoma of the cervix Clin Cancer Res 2004 Dec 15 10 24 8405 8412 10.1158/1078-0432.CCR-03-0135 15623619

[B17] Beasley NJ Leek R Alam M Turley H Cox GJ Gatter K Hypoxia-inducible factors HIF-1alpha and HIF-2alpha in head and neck cancer: relationship to tumor biology and treatment outcome in surgically resected patients Cancer Res 2002 May 1 62 9 2493 2497 11980639

[B18] Fillies T Werkmeister R van Diest PJ Brandt B Joos U Buerger H HIF1-alpha overexpression indicates a good prognosis in early stage squamous cell carcinomas of the oral floor BMC Cancer 2005 5 84 10.1186/1471-2407-5-84 16035955 PMC1190162

[B19] Shaida N Chan P Turley H Jones CM Kanga S Ritchie RW Nuclear localization of factor inhibitor hypoxia-inducible factor in prostate cancer is associated with poor prognosis J Urol 2011 Apr 185 4 1513 1518 10.1016/j.juro.2010.12.001 21334674

[B20] Simon J Comperat E Beckendorf P Bey P Mazeron J Jaillon P Valeurs prédictives des expressions HIF-1 alpha et CAIX par les adenocarcinomes de la prostate traités par irradiation exclusive. Etude ancillaire du protocole GETUG 06 Cancer Radiother 2007 11 Abstract P083 414

[B21] Carmeliet P Dor Y Herbert JM Fukumura D Brusselmans K Dewerchin M Role of HIF-1alpha in hypoxia-mediated apoptosis, cell proliferation and tumour angiogenesis Nature 1998 Jul 30 394 6692 485 490 10.1038/28867 9697772

[B22] Vaughan MM Toth K Chintala S Rustum YM Double immunohistochemical staining method for HIF-1alpha and its regulators PHD2 and PHD3 in formalin-fixed paraffin-embedded tissues Appl Immunohistochem Mol Morphol 2010 Jul 18 4 375 381 10.1097/PAI.0b013e3181d6bd59 20216402 PMC3215297

[B23] Pipinikas CP Carter ND Corbishley CM Fenske CD HIF-1alpha mRNA gene expression levels in improved diagnosis of early stages of prostate cancer Biomarkers 2008 Nov 13 7 680 691 10.1080/13547500802591992 19096962

[B24] Di Lorenzo G Tortora G D’Armiento FP De Rosa G Staibano S Autorino R Expression of epidermal growth factor receptor correlates with disease relapse and progression to androgen-independence in human prostate cancer Clin Cancer Res 2002 Nov 8 11 3438 3444 12429632

[B25] Vuky J Porter C Isacson C Vaughan M Kozlowski P Picozzi V Phase II trial of neoadjuvant docetaxel and gefitinib followed by radical prostatectomy in patients with high-risk, locally advanced prostate cancer Cancer 2009 115 4 784 791 10.1002/cncr.24092 19130458

[B26] Gyftopoulos K Vourda K Sakellaropoulos G Perimenis P Athanasopoulos A Papadaki E The angiogenic switch for vascular endothelial growth factor-A and cyclooxygenase-2 in prostate carcinoma: correlation with microvessel density, androgen receptor content and Gleason grade Urol Int 2011 87 4 464 469 10.1159/000329289 21912077

[B27] Jackson MW Bentel JM Tilley WD Vascular endothelial growth factor (VEGF) expression in prostate cancer and benign prostatic hyperplasia J Urol 1997 Jun 157 6 2323 2328 10.1016/S0022-5347(01)64774-8 9146664

